# Assessing Executive Functions in Preschoolers Using Shape School Task

**DOI:** 10.3389/fpsyg.2016.01489

**Published:** 2016-09-27

**Authors:** Marta Nieto, Laura Ros, Gloria Medina, Jorge J. Ricarte, José M. Latorre

**Affiliations:** Department of Psychology, Faculty of Medicine and Research Institute of Neurological Disabilities, University of Castilla-La ManchaAlbacete, Spain

**Keywords:** executive functions, inhibition, switch, working memory, general cognitive abilities, Shape School, preschoolers

## Abstract

Over the last two decades, there has been a growing interest in the study of the development of executive functions (EF) in preschool children due to their relationship with different cognitive, psychological, social and academic domains. Early detection of individual differences in executive functioning can have major implications for basic and applied research. Consequently, there is a key need for assessment tools adapted to preschool skills: Shape School has been shown to be a suitable task for this purpose. Our study uses Shape School as the main task to analyze development of inhibition, task-switching and working memory in a sample of 304 preschoolers (age range 3.25–6.50 years). Additionally, we include cognitive tasks for the evaluation of verbal variables (vocabulary, word reasoning and short-term memory) and performance variables (picture completion and symbol search), so as to analyze their relationship with EFs. Our results show age-associated improvements in EFs and the cognitive variables assessed. Furthermore, correlation analyses reveal positive relationships between EFs and the other cognitive variables. More specifically, using structural equation modeling and including age direct and indirect effects, our results suggest that EFs explain to a greater extent performance on verbal and performance tasks. These findings provide further information to support research that considers preschool age to be a crucial period for the development of EFs and their relationship with other cognitive processes.

## Introduction

Executive functions (EF) comprise a family of mental processes associated with the functions of the prefrontal cortex (Müller and Kerns, 2015). More specifically, they refer to high-level cognitive processes oriented toward reactive inhibition and the regulation of goal achievement behavior (Carlson et al., 2013). A three-component structure is the most widely proposed: *inhibition* (suppression of prepotent or affectively driven behaviors); *working memory* ([WM] to hold information active in mind and to mentally work with that information as a platform for guiding our behavior); and *shifting* (switching flexibly between tasks or mental sets) (Miyake et al., 2000). However, a number of authors suggest that while shifting and working memory have a specific component in addition to the common EF component, inhibition is thought to be contained completely within the common EF ability (Miyake and Friedman, 2012). In other words, individual differences in inhibition are explained by what is common to all three EF and, hence, there is no inhibiting-specific factor (Friedman et al., 2008, 2011). Some studies with preschoolers have supported a unidimensional construct of executive functioning (Fuhs and Day, 2011; Wiebe et al., 2011). However, others suggest the presence of multiple EF even in the early years (Usai et al., 2014; Howard et al., 2015; Skogan et al., 2016), hence the debate on this matter remains open as methodological differences have complicated the comparison of competing theoretical models of EF in this age group (van der Ven et al., 2013; Wasserman and Wasserman, 2013).

A review of the literature on EF from birth to 5 years uses an attentional framework to showcase the hierarchical development of these skills (Miyake et al., 2000; Garon et al., 2008). Although some basic skills are exhibited across the second year of life (Garon et al., 2008, 2014; Wiebe et al., 2010; Miller and Marcovitch, 2015), the preschool years have been identified as a crucial period in the emergence and development of EF (Espy, 1997; Carlson, 2005; Garon et al., 2008; Howard et al., 2015), which continue to develop across middle childhood and adolescence (Best et al., 2009). More specifically, Garon et al. (2008) find that basic processes, such as delaying gratification and holding information in mind, emerge in the first 3 years of life. Next, elementary forms of the core components appear to gradually integrate more complex, aspects of EF (WM, inhibition and shifting). Finally, the EF which develops fully after the preschool period, such as planning or conceptual reasoning, build on previously acquired skills. Consequently, it has been suggested that the development of EF during the preschool years results in qualitative changes in cognitive functions, while later advances are mainly related to aspects of a more quantitative nature (Best and Miller, 2010).

Most studies with young children have demonstrated age-related differences in executive performance (Diamond et al., 2002; Edgin et al., 2008), coinciding with research on brain maturation in childhood, particularly the development of the prefrontal cortex (e.g., Gogtay et al., 2004; Rubia, 2013). Espy et al. (1999) administered a battery of EF tasks to children aged 23–66 months. They found significant age differences on all of the EF tasks, including inhibition, set-shifting, and WM tasks. In a later study with children aged 30–60 months, Espy et al. (2001) concluded that WM-related tasks were most sensitive to age effects. Carlson (2005) provided cross-sectional data on 602 preschool children, suggesting age-associated improvements in executive performance, which might also be modulated by task difficulty. Garon et al. (2014) recently presented a new EF battery (*Preschool Executive Function Battery:* hide and seek [WM], tricky box [inhibition], and flap book [shifting]) that is sensitive to age differences between preschoolers from 1.5 to 5 years and provides partial support for the hierarchical model of EF development. More specifically, research based on the Shape School task (Espy, 199) reports similar findings. For example, Espy et al. (2001) selected the control and inhibition Shape School conditions, finding that children of 36 months performed worse than older preschoolers. In an analysis of 219 children (young, middle, and older preschooler) who completed the Shape School, Espy et al. (2004) found that, generally speaking, the time to complete the four conditions varied by age group, but not the number of stimuli correctly named. For the switch condition, the middle age group took longer on average to complete the condition than the younger children, but completed the condition in less time than older children. In this line, the work by Pritchard and Woodward (2011) provides evidence to support the predictive utility of the Shape School task with preschoolers. Firstly, the authors analyzed the Shape School performance of typically developing children on the control, inhibit, and switch conditions at age 4 years. The results showed that while performance on control and inhibition was adequate, the study sample showed a significant decrement for the switch condition. Secondly, the task performance of the full-term and very preterm children was compared. The results showed the preterm group scored lower on all three conditions. Finally, the authors identified associations between the Shape School task and neurodevelopmental functioning and later academic achievement.

With respect to other domains, the EF has been considered essential for physical and mental health, academic achievement and cognitive, social, and psychological development (Diamond, 2013, for a review; Loe et al., 2015; Ng et al., 2015). Executive deficits have been associated with neurodevelopmental disorders (de Vries and Geurts, 2015; Ezpeleta and Granero, 2015; Niloufar and Reza, 2015) and different types of psychopathology (McNamara et al., 2014; Myers and Wells, 2015).

Over the last two decades, there has been a growing interest in the study of the development of EF during early childhood and assessment tools have been designed. Taking into account the impulsive behaviors and attentional, motor and linguistic limitations of children, tasks must be adapted to their abilities. They should be based, for example, on everyday activities, such as reward expectancy (Kochanska et al., 2000), imitation tasks (Diamond and Taylor, 1996) or searching for hidden objects (Espy et al., 1999); and on familiar notions, such as simple shapes, animals, everyday objects or colors (Espy, 1997; Prevor and Diamond, 2005).

The Shape School Task (Espy, 1997) was developed to assess EF in preschoolers. This task provides separate assessment of inhibition and switching in young children. In this line, it has been suggested that cognitive processes differ maturationally and contribute uniquely to executive skill development. Consequently, according to Espy (1997), Shape School is sensitive to age-related improvements in inhibition and switching processes, making it a useful tool for evaluating the emergence and development of EF. For example, the first study by Espy (1997) found that 4-year-old children inhibited more efficiently than 3-year-old; in contrast, switching efficiency improved between 4 and 5 years of age. Although these findings suggest that the developmental pattern of inhibition and switching abilities varies according to age, these conclusions must be treated cautiously since the switch condition was not administered to the children less than 48 months of age. Furthermore, bearing in mind that Shape School is a verbal naming task, higher verbal attitude might be associated with differences in performance across age groups (Espy, 1997; Espy et al., 2006). Moreover, it has also been suggested that children’s use of language may facilitate their performance on EF task (e.g., Kirkham et al., 2003; Brace et al., 2006). Finally, adequate psychometric properties and convergent validity have been identified for the Shape School Task (Espy et al., 2006).

The present study aims to follow the line of research based on the development of EF in preschoolers using Shape School (e.g., Espy, 1997; Espy et al., 2001, 2004; Pritchard and Woodward, 2011). We consider it important to develop versions of Shape School adapted to different countries. Several studies have shown that the educational characteristics of countries influence the cognitive and language development of the children (e.g., Cryer et al., 1999). For example, Montie et al. (2006) found that increased adult-child interaction in preschool is related to better language scores in countries that have less adult-centered teaching or activities that require group response, and poorer language scores in countries that have more adult-centered teaching or activities that require group response. Consequently, it would be expected for the educational characteristics of a certain country to influence performance on Shape School. For this reason, using a larger study sample than in previous works, we have developed an adapted version of the Shape School for use in Spanish preschool populations. Our work was motivated by the growing theoretical and empirical interest in the study of the EF in preschool children, stemming from their impact on the development of various cognitive, emotional, social and academic abilities. Furthermore, the recognition of the stages that are more sensitive to executive development and the associated variables is a primary objective for the implementation of social and educational programs, the design of methods to identify high-risk populations and the creation of interventions aimed at specific early developmental problems. Thus, given the interest in this subject, our work focuses on two main aims. The first aim is to analyze the development of EF in preschoolers and the age-associated differences in inhibition, switch, and inhibition-switch using the Shape School task and, additionally to analyze WM using Word Span Backward (WSB). The second aim focuses on studying the relationship between EF and other cognitive abilities (vocabulary tests, symbol searches, word reasoning and picture completion, taken from the Wechsler Preschool and Primary Scale of Intelligence, and Short-Term Memory) so as to analyze to what extent of the possible relationship between executive performance in preschool years and the development of other cognitive capacities (verbal and performance capacities).

## Materials and Methods

### Participants

Preschool age in Spain refers to the developmental period from 3 to 6 years. At an educational level, this age corresponds to the second stage of infant education. This non-compulsory stage aims to contribute to children’s physical, affective, social and intellectual development (Royal Decree, 1630/2006, of 29 December).

Typically developing preschoolers from six public and two grant-aided schools in an urban area of Spain took part in this study. Teaching at all the schools is conducted in Spanish, although pupils have two hourly classes of English per week. All participants were recruited by the school counseling teams. A total of 97% of the children in the schools took part. The remaining 3% did not take part due to lack of parental consent. The sample comprised 304 Spanish Caucasian participants aged 3–6 years, (54.9% girls; ages 3.25-6.50 years old; *M* = 4.66, *SD* = 0.88). The preschoolers were grouped by age: 3–4 years (*N* = 79; 58.2% girls; ages 3.25–3.92 years old; *M* = 3.61, *SD* = 0.17); 4–5 years (*N* = 113; 52.2% girls; ages 4–4.92 years old; *M* = 4.39, *SD* = 0.27); and 5–6 years (*N* = 112; 55.4% girls; ages 5–6.50 years old; *M* = 5.68, *SD* = 0.39). All the children were from high and medium SES families, with annual incomes ranging from € 25,000 to € 56,000.

### Measures

#### Shape School

The Shape School task (Espy, 1997), in a version adapted for Spanish populations by Ros et al. (2011) was used to assess EF. Shape School is an individually administered task in a storybook format. It includes four experimental conditions, comprising 15 stimuli presented in a fixed order: control, inhibition, switch and inhibition-switch. The story begins by presenting a school where the children are colorful circles and squares. In the control condition the child is told that the pupils’ names are the figures’ colors (8 SQUARES [red = 3; blue = 3; and yellow = 2] and 7 CIRCLES [red = 3; blue = 1; and yellow = 3]). The story continues with the pupils lining up to go into school. The participant is instructed to name the stimuli by their color as fast as possible without making any errors. This condition serves two purposes: to measure base line naming speed and to determine the relationship between the properties of the stimulus and the participant’s responses. In the inhibition condition the figures have two facial expressions, happy and sad, depending on whether the pupils are ready for lunch (happy) or no (sad). The participant is instructed to name only the figures with a happy expression and not to mention those with a sad face (10 happy faces [6 SQUARES: red = 2; blue = 2; and yellow = 2//4 CIRCLES: red = 2; blue = 1; and yellow = 1] and 5 sad faces: [2 SQUARES: red = 1; and blue = 1//3 CIRCLES: red = 1; and yellow = 2]). This condition is designed to measure an inhibitory process, i.e., a suppressive response.

The control and inhibition conditions are administered to all preschoolers. The following two are administered only to those of four and over since they use the principle of shape, in addition to color (Espy, 1997). In the switch condition, as in the control condition, all the pupils have neutral faces. Participants are told that the pupils are going to listen to a story and are instructed to name the stimuli in order, depending on whether they are wearing a hat (name the shape) or not (name the color) (7 hatless stimuli [5 SQUARES: red = 2; blue = 2; and yellow = 1//2 CIRCLES: red = 1; and yellow = 1] and 8 hatted stimuli [3 SQUARES: red = 1; blue = 1; and yellow = 1// 5 CIRCLES: red = 2; blue = 1; and yellow = 2]). This condition measures cognitive switching as participants have to simultaneously use the principles of shape and color. Finally, the inhibition- switch condition includes pupils with happy or sad faces and with or without hats. The participant is told that the pupils are ready for a party. The preschooler is then asked to name the happy-faced pupils by color if hatless or by shape if hatted and is specifically instructed not to mention the stimuli with sad faces (10 happy faces and 5 sad faces: 8 hatless stimuli [5 SQUARES: red = 1; blue = 3; and yellow = 1// 3 CIRCLES: red = 1; and yellow = 2] and 7 hatted stimuli [3 SQUARES: red = 2; and yellow = 1//4 CIRCLES: red = 2; blue = 1; and yellow = 1]). This condition determines suppressive response and task switching.

There are practice examples for all conditions to ensure the preschoolers understand the instructions. The scores achieved in each condition give an efficiency value which is calculated as follows: (Efficiency = [number of correct answers – number of errors]/total time in seconds for each conditions).

The study by Espy et al. (2006) demonstrated adequate reliability indices for the Shape School task. Cronbach’s alpha coefficients computed with the responses to each of the stimuli within each condition revealed adequate association in the executive conditions, inhibition (α = 0.71), switch (α = 0.80), and inhibition-switch (α = 0.74). In control condition (α = 0.56), the coefficient likely was attenuated due to the high level of naming accuracy in this very simple condition.

#### Word Span

This test is the Spanish version of the procedure developed by Thorell and Wählstedt (2006), based on the Digit Span subtest from the Wechsler Intelligence Test for Children-3rd edition (WISC-III; Wechsler, 1991). According to Thorell and Wählstedt (2006), test–retest reliability was adequate, *r* = 0 67, *p* < 0.001, using the scores from 24 children between the ages of 4–5 years tested 2 weeks apart.

Word Span includes two tasks, Word Span Forward (WSF) and Word Span Backward (WSB) which measure respectively the processes of short-term maintenance of information and the handling of information in the working memory. We decided to use words rather than digits due to the age of the participants. The words were selected from among familiar concepts and adapted to the development of preschool vocabulary (e.g., cat, tree, and milk). The tasks are independently and individually administered. In the WSF task, the examiner reads a sequence of common words of increasing length, which the participant is asked to repeat from memory in the same order. The length of the sequence varies between two and seven words (six sequences) and two different sequences are presented for each length (12 different sequences). The task ends when the participant is unable to remember either of the two same-length sequences. The WSB task has the same characteristics as the previous one but the process is different as the participant is asked to repeat the words in the reverse order from the spoken sequence. This task also finishes when the participant is unable to remember either of the two same-length sequences. In each task a point is scored for each correctly remembered sequence.

The procedure used by Thorell and Wählstedt (2006), calculated the mean value of the two Word Span conditions as a joint measure of working memory. In contrast, for the current study, WSF and WSB were analyzed as independent tasks for measuring short-term memory and working memory, respectively, since several authors have indicated that WSB is the most appropriate measure of working memory capacity (Gathercole et al., 2004; Diamond, 2013).

#### Wechsler Preschool and Primary Scale of Intelligence

In order to measure cognitive verbal and performance variables, we used the direct scores on the subtests of the Spanish version of the Wechsler Preschool and Primary Scale of Intelligence (WPPSI-III; Wechsler, 2009), which were all administered individually.

##### Vocabulary

A verbal test, which measures the child’s ability to form verbal concepts and word comprehension level. It also measures general knowledge, learning potential and level of language development. It includes visual items where the child names pictures in a stimulus book. The preschooler verbally describes first common objects and then concepts requiring greater abstraction. The test comprises 25 items: 5 picture items and 20 verbal items. For the picture items, the child names the pictures shown in the stimuli book. For the verbal items, the child defines words that are read aloud by the examiner. The maximum score is 43 points. The test is terminated when the child makes five consecutive mistakes. Timing is not recorded. The reliability coefficient of the vocabulary test is α = 0.76.

##### Symbol search

Information processing speed test, which also measures short-term visual memory, eye-hand coordination, cognitive flexibility, visual discrimination and concentration. It can also relate to auditory comprehension, perceptual organization and planning and learning abilities. The test consists of 50 items. The task consists of determining whether a target symbol matches any symbols in a search group of three. The child then crosses out the matching symbol or the word NO if the target symbol does not appear. The administration time is 120 s. The direct score is reached by subtracting the number of mistakes from the number of correct answers. The maximum score is 50 points. The reliability coefficient of the symbol search is α = 0.82.

##### Word reasoning

A verbal test where the preschooler has to identify a series of common concepts being described using key words or clues. Based on short clues provided by the examiner, the child has to guess the different items. As the test progresses, the level of difficulty increases (e.g., “a drink that comes from cows” or “the noise we make with our mouth…when something funny happens”). It relates to tasks designed to assess verbal reasoning, verbal comprehension, analogical and general reasoning ability, the ability to integrate and synthesize different types of information, verbal abstraction, domain knowledge and the ability to generate alternative concepts. The test comprises 28 items, plus two trial items. The maximum score is 28 points. Timing is not recorded. The reliability coefficient for the word reasoning task is α = 0.84.

##### Picture completion

A manipulative test, which measures visual perception and organization, concentration and visual recognition of the essential details of objects. The test consists of 32 items, plus two trial items. For all items, the examiner shows the child an incomplete picture and the child is asked to find the missing part, which they then have to name or indicate. The test is terminated when the child makes five consecutive mistakes. If no response is given within 20 s or a mistake is made in less than 20 s, the next item is administered if the termination criterion has not been met. The total direct score is 32 points. The reliability coefficient for the picture completion task is α = 0.84.

### Procedure

The schools participating in the study invited the preschoolers’ parents or legal guardians to attend an informational meeting. Informed consent was obtained from those who wanted their children to take part in the study.

Data collection was conducted by nine experimenters. Participants were individually assessed during the school day, outside the classroom but within the school building. The assessment was conducted in two sessions of around 90 min. The WPPSI-III was administered in the first session and the Word Span tests and Shape School task in the second. After finishing the task, the children were rewarded with a sticker and then returned to class.

## Results

### Main Analysis

**Table 1** shows the main descriptive results.

**Table 1:** Means and standard deviations for the main variables in the study by age group.

lVariable	3–4 years (*n* = 79)	4–5 years (*n* = 113)	5–6 years (*n* = 112)	η_p_^2^	Cohen’s *d*
l**ShS. Control**					
lTime^a^	30.56 (15.55)	25.57 (13.11)	19.08 (6.13)	0.13^∗∗^	
lCorrect answers	14.30 (2.10)	14.71 (1.59)	14.96 (0.18)	0.03^∗^	
lEfficiency	0.54 (0.21)	0.66 (0.22)	0.83 (0.20)	0.22^∗∗^	
l**ShS. Inhibit**					
lTime	46.86 (19.67)	35.35 (17.08)	25.32 (10.65)	0.22^∗∗^	
lCorrect answers	11.67 (4.01)	13.45 (3.50)	14.49 (1.09)	0.12^∗∗^	
lEfficiency	0.25 (0.16)	0.43 (0.22)	0.62 (0.23)	0.31^∗∗^	
l**ShS. Switch**	–				
lTime		46.54 (24.06)	35.29 (13.12)		0.58^∗∗^
lCorrect answers		11.99 (3.33)	13.42 (2.57)		-0.48^∗∗^
lEfficiency		0.24 (0.19)	0.39 (0.17)		-0.81^∗∗^
l**ShS. Inhibition-switch**	–				
lTime		49.16 (19.10)	39.72 (2.54)		0.58^∗∗^
lCorrect answers		10.10 (3.92)	12.29 (3.26)		-0.61^∗∗^
lEfficiency		0.12 (0.19)	0.25 (0.20)		-0.66^∗∗^
l**Word Span**					
lWSF	3.73 (1.28)	4.37 (1.20)	4.89 (1.06)	0.13^∗∗^	
lWSB	0.27 (0.74)	1.02 (1.21)	2.09 (1.17)	0.31^∗∗^	
l**WPPSI-III**					
lVocabulary	12.82 (3.47)	14.09 (4.93)	20.15 (6.47)	0.27^∗∗^	
lSymbol search	4.04 (3.97)	8.11 (6.46)	14.74 (8.35)	0.42^∗∗^	
lWord reasoning	9.22 (4.83)	13.83 (5.61)	19.40 (4.40)	0.39^∗∗^	
lPicture completion	9.34 (5.02)	13.03 (5.27)	19.69 (5.62)	0.38^∗∗^	

*ShS, Shape School; WSF, word span forward; WSB, word span backward. ^a^Time in seconds for the four Shape School conditions. ^∗^*p* < 0.050; ^∗∗^*p* < 0.001.*

ANOVAs were conducted to analyze the effect of age on the variables evaluated using Shape School and WSB. The results show statistically significant differences by age for all the variables: All *p* < 0.001: WSB *F*(2,302) = 67.29, *p* < 0.001, η_p_^2^ = 0.31; Shape School control efficiency *F*(2,302) = 42.51, *p* < 0.001, η_p_^2^ = 0.22; Shape School inhibit efficiency *F*(2,302) = 68.73, *p* < 0.001, η_p_^2^ = 0.31. Furthermore, the *post hoc* Scheffé analyses found significant differences between the three age groups (all *p ≤* 0.001). Finally, mean comparisons were performed with the participants aged over 4 for the last two Shape School conditions. The results show statistically significant differences between the 4–5 and 5–6 age groups on the switch efficiency [*t*(215) = -5.90, *p* = 0.001, Cohen’s *d* = -0.81] and inhibition-switch efficiency [*t*(215) = -5.05, *p =* 0.001, Cohen’s *d* = -0.66].

### Correlation Analysis

In all the correlation analyses, we used the efficiency parameter for each of the Shape School conditions. **Table 2** shows the correlations across the study variables for 3–4 years age group. The Shape School conditions (control and inhibition), WSB (WM), the different cognitive tasks assessed using WPPPI-III (vocabulary, symbol search, word reasoning, and picture completion), and WSF (short-term memory) were all positively correlated. **Table 3** shows the correlations for the children aged over 4 years (4–5 and 5–6 years age groups). It can be seen that in the younger group, all the variables assessed (EF, verbal and performance variables, and short-term memory) were highly and positively correlated.

**Table 2:** Correlations between study variables for participants under 4 years (*n* = 79).

	1	2	3	4	5	6	7	8
(1) ShS. Control efficiency	–							
(2) ShS. Inhibit efficiency	0.56^∗∗^	–						
(3) WSB	0.50^∗^	0.28^∗^	–					
(4) Vocabulary	0.21^∗^	0.26^∗^	0.24^∗^	–				
(5) SS	0.39^∗∗^	0.54^∗∗^	0.35^∗∗^	0.33^∗∗^	–			
(6) Word reasoning	0.37^∗∗^	0.50^∗∗^	0.44^∗∗^	0.42^∗∗^	0.45^∗∗^	–		
(7) PC	0.13	0.34^∗∗^	0.04	0.30^∗∗^	0.25^∗^	0.30^∗∗^	–	
(8) WSF	0.14^∗^	0.18^∗^	0.37^∗∗^	0.33^∗∗^	0.28^∗^	0.37^∗∗^	0.24^∗^	–

*ShS, Shape School; WSB, word span backward; SS, symbol search; PC, picture completion; WSF, word span forward. ^∗^*p <* 0.050; ^∗∗^*p <* 0.001.*

**Table 3:** Correlations between study variables for participants aged over 4 (*n* = 225).

	1	2	3	4	5	6	7	8	9	10
(1) ShS. Control efficiency	–									
(2) ShS. Inhibit efficiency	0.58^∗^	–								
(3) ShS. Switch efficiency	0.44^∗^	0.55^∗^	–							
(4) ShS. Inhibit-switch efficiency	0.33^∗^	0.49^∗^	0.63^∗^	–						
(5) WSB	0.34^∗^	0.46^∗^	0.46^∗^	0.41^∗^	–					
(6) Vocabulary	0.48^∗^	0.46^∗^	0.49^∗^	0.38^∗^	0.53^∗^	–				
(7) SS	0.47^∗^	0.54^∗^	0.51^∗^	0.42^∗^	0.51^∗^	0.64^∗^	–			
(8) Word reasoning	0.28^∗^	0.42^∗^	0.44^∗^	0.38^∗^	0.46^∗^	0.57^∗^	0.46^∗^	–		
(9) PC	0.25^∗^	0.45^∗^	0.41^∗^	0.37^∗^	0.42^∗^	0.49^∗^	0.40^∗^	0.60^∗^	–	
(10) WSF	0.32^∗^	0.36^∗^	0.35^∗^	0.36^∗^	0.49^∗^	0.45^∗^	0.36^∗^	0.50^∗^	0.31^∗^	–

*ShS, Shape School; WSB, word span backward; SS, symbol search; PC, picture completion; WSF, word span forward. ^∗^*p <* 0.001.*

### Structural Equation Modeling

Structural equation models (SEM) were tested using the AMOS 18.0 software (IBM; Madrid, Spain; Arbuckle, 2009) to estimate the relationship between EF variables and WIPPSI variables, including age direct and indirect effects. SEMs were conducted using IBM-SPSS AMOS-19. In the SEMs, maximum likelihood was used to estimate all model parameters. In order to evaluate the model fit, we used the comparative fit index (CFI; Bentler, 1989; Hu and Bentler, 1999), the Tucker and Lewis index (TLI; Tucker and Lewis, 1973), and the root-mean-square error of approximation (RMSEA; Browne and Cudeck, 1993). According to Bentler (1990), CFI and TLI values greater than 0.90 are indicative of an acceptable fit. As regards RMSEA, values below 0.05 represent a good fit (Byrne and Campbell, 1999).

**FIGURE 1:**
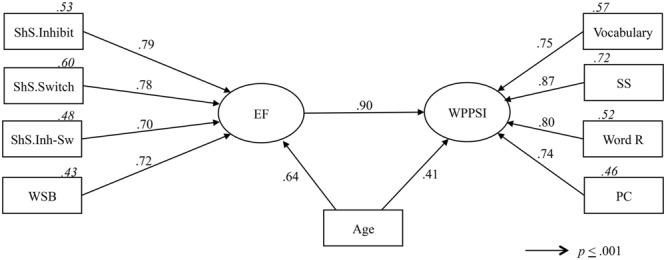
**Structural equation model.** The Shape School conditions are based on the efficiency score; ShS, Shape School; Inh-Sw, inhibit-switch; WSB, word span backward; EF, executive functions; SS, symbol search; Word R, word reasoning; PC, picture completion. Numbers in italics represent squared multiple correlations.

The SEM model was calculated using only the sample of children older than 48 months (4–5 and 5–6 years age groups), since the children in the 3–4 years age group were unable to complete the final two Shape School conditions (switch and inhibition-switch). In this model, the scores obtained on the Shape School inhibition, switch, inhibition-switch conditions and the word span backward variable were considered, thus forming a latent variable of a unidimensional construct of EF. **Table 4** shows the goodness of fit statistics. **Figure 1** shows the estimated parameters (presented as standardized). According to these statistics, 30% of the variance of the WIPPSI latent variable (comprising vocabulary, symbol search, word reasoning, and picture completion observed variables), is explained by the EF latent variable while 16.8% is explained by the age variable. Results also show that the standardized indirect effect of age on WIPPSI latent variable through EF latent variable is.35 (*p* = 0.009).

**Table 4:** Fit statistics for structural equation model (*n* = 225).

Variable	df	χ^2^	*p*	RMSEA	TLI	CFI
EF-WIPPSI	22	30.32	0.111	0.04	0.98	0.99

*EF, executive functions; RMSEA, root-mean-square error of approximation; TLI, Tucker-Lewis Index; CFI, comparative fit index.*

## Discussion

The first aim of this study is to analyze the development of EF during the preschool years. As our findings show, basic forms of inhibition, switch and WM are present in preschoolers and age-associated improvements in performance can be observed. Regarding the Shape School task, our results are similar to those of the original study by Espy (1997), and those of further research conducted using the task (Espy et al., 2001, 2004; Pritchard and Woodward, 2011; Nieto et al., submitted). Detailed analysis of the Shape School conditions shows that the highest efficiency is found in the control condition, in which all three groups scored near the maximum for correctly named stimuli while latency decreased according to age. However, the demands of control are not truly EF, hence it has been suggested that this condition be considered an indicator of baseline capacity of information processing (Pritchard and Woodward, 2011). Across all age groups, for the inhibition condition, accuracy in naming stimuli progressively increased and latency decreased according to age. The same performance pattern can be seen for the switch and inhibition-switch conditions in the 4–5 and 5–6 years age groups. Interestingly, across all age groups, accuracy decreases and latency rises as task difficulty increases. These findings are consistent with the literature on early executive control when inhibitory and set shifting skills are believed to follow different developmental trajectories, with the latter still largely immature by early school age (Espy and Bull, 2005; Pritchard and Woodward, 2011). Indeed, as noted by Espy (1997), the last two Shape School conditions are only administered to children older than 48 months because these conditions also utilize the principle of shape, in addition to color, which younger children may not process automatically. Regarding the WSB task, the results showed significant differences between groups with an increase in WM capacity by age being observed. These results coincide with previous studies using span tests with preschoolers (Müller et al., 2012; Nieto et al., submitted). However, it has been noted that preschoolers, and particularly younger preschoolers, have difficulties in understanding the concept of backward in the WM task (Bull et al., 2008). Consequently, the performance of the study sample, especially those of the 3–4 age group, could be influenced by the demands of the task.

During the preschool years, significant changes occur in other capacities, such as attention, language, or motor and ocular factors, whose development may be associated with the EF (Miyake et al., 2000; Newsham et al., 2007). Consequently, the second aim of this study was to analyze the relationship between performance on Shape School and WSB and different cognitive capacities. Our results show a positive relationship between the Shape School and WSB and verbal and performance variables. Previous studies consider attention to be an essential variable in the development of the EF (Garon et al., 2008). In fact, differences in attentional processes during early childhood predict later capacity of response inhibition (Sethi et al., 2000). Moreover, manipulation of attention in set-shifting sets has a significant effect on the performance of children aged from 2 months to 4 years (Kirkham et al., 2003). Finally, similar results on attention control tasks have been found to differentiate preschoolers with low and high working memory span (Espy and Bull, 2005). Our results show that, broadly speaking, the ability to hold a larger number of items in mind, as assessed by WSF and WSB, was related to more efficient performance on Shape School. In this line, high correlations between Shape School and WM were found in inhibition across the whole study sample and in switch for the children over 48 months of age. These abilities have the highest working memory load, as identified in both Shape School (Espy et al., 2006) and other assessment methods (Dick, 2014).

Regarding verbal ability, EF has been related to language ability in preschoolers (e.g., Carlson and Moses, 2001; Hughes and Ensor, 2007), and correlations between EF and language skills are frequently reported (e.g., Carlson et al., 2005; Gooch et al., 2016). As previously mentioned, Espy et al. (2006) highlighted the verbal load of Shape School and our results show that, regardless of condition, verbal aptitudes were related to efficiency. Additionally, WM capacity was also related to verbal aptitude, and in the present study it can be seen that vocabulary, which includes a child’s ability to form verbal concepts, word comprehension level, general knowledge, learning potential and level of language development, and the verbal reasoning subtest, which includes verbal reasoning, comprehension and abstraction, and the ability to synthesize and generate alternative concepts, were positively related to WM capacity. Finally, further variables, such as ocular control and motor skills, have been associated with performance in Shape School (Pitcher et al., 2003; Newsham et al., 2007). Regarding these aptitudes, Pritchard and Woodward (2011) examined motor skills and ocular control and their association with performance on Shape School. Their results showed that children with motor disorders exhibited generally less efficient performance, while deficits in ocular control were specifically associated with inhibition. In this line, future works could focus on assessing these aptitudes in Shape School, using specifically designed tasks and procedures (e.g., Palisano et al., 1997; Shah et al., 2006).

With respect to maturational changes occurring during brain development which coincides with marked improvements in cognitive, motor and perceptual abilities in young children (see Casey et al., 2005, for a review), using structural equation modeling, we conducted a more exhaustive analysis of the relationship between EF and verbal and performance variables, including age direct and indirect effects. Our results show that EF (inhibition, switch, inhibition-switch, and WM) seems to explain performance on verbal and manipulation tasks to a greater extent than the age variable. Nevertheless, it should be noted that age also presents a significant indirect effect on verbal and performance variables through EF. Viterbori et al. (2012) suggest that more efficient EF processing may promote better language skills in typical language development in young children. More specifically, their findings showed that inhibition predicted intelligibility and phonological accuracy, whereas morphological and syntactic abilities were associated with inhibitory control and cognitive flexibility. Furthermore, the essential role of working memory capability in integrating cognition and language has also been highlighted (Belacchi et al., 2014). Schoemaker et al. (2013) recently found that lower EF levels in a preschool sample were associated with attentional problems and higher levels of hyperactive/impulsive behaviors. In young schoolchildren, EF may allow using processes that are necessary for knowledge acquisition such as remembering instructions and attending to important features of the lesson, staying on task, and dealing with abstract concepts and symbols (Best et al., 2009). EF also predicts later performance in mathematics and literacy in children and adolescents (e.g., Best et al., 2011; Neuenschwander et al., 2012). In addition, from a clinical perspective the role of EF has been highlighted, for example, in predicting the development of play skills for verbal preschoolers with autism spectrum disorders (Faja et al., 2016). Consequently, knowledge of EF and their influence on and relationship with other key abilities may lead to better methods of understanding factors influencing early development.

This work has a number of limitations. First, we did not specifically assess the neurocognitive and behavioral changes and the improvements associated with education from 3 to 6 years. The second limitation is related to the assessment of WM in our study sample, since the youngest children had difficulties as a consequence of the demands of the task. An alternative to span tasks could be provided by the Missing Scan Task, which has recently been shown to be a suitable measure for assessing WM in preschool children as young as 3 years of age (Roman et al., 2014). The third limitation refers to the transversal nature of the study. Longitudinal studies provide information on the progressive development of EF during the preschool ears (Hughes and Ensor, 2011; Visu-Petra et al., 2015), hence, longitudinal analysis of the specific components of EF (simple and complex forms of EF) from 3 to 6 years, could further the research significantly. For example, the study by Garon et al. (2014) suggest that inhibition and WM have different developmental trajectories in the preschool age. Consequently, a larger number of longitudinal studies are needed to confirm the findings of the present work. Lastly, as our study was limited to a normative sample of preschoolers, an interesting line of research would be to focus on the study of EF, including clinical samples, to determine the baseline of their development and to compare performance across different groups, given the physical, psychological, social and academic implications of executive deficits from an early age.

This work provides further information on the development of EF in preschoolers and the age-associated improvements, using Shape School as the main task. More specifically, increases in inhibit and switch are observed from 3 to 5 years, as well as early emergence of basic WM skills. The findings also show the importance of assessing various aspects of executive performance in preschoolers in order to identify specific strengths and deficits in early development, because, including age direct and indirect effects, EF have been shown to be variables which explain performance on other cognitive tasks. Finally, we suggest that the Spanish version of Shape School (Ros et al., 2011) could be a useful tool for the assessment of EF from 3 to 6 years. It is simple and quick to administer and has adequate psychometric properties (Espy et al., 2006). Consequently, it may be considered a valid tool to be administered by educators and counselors in school settings, complementing assessment of verbal variables and performance. As we know, the correct development of executive processes during the preschool years allows children to successfully adapt to their environment. It acts as a springboard for children to achieve more mature behaviors in later life stages, such as the regulation of behavior depending on the demands of different settings and the acquisition of social skills for interacting with peers, adapting their behavior to the norms of the social system in which they are involved (e.g., turn taking in class) (see Stelzer et al., 2011, for a review). Consequently, we consider that more in-depth study of the early development of EF, using appropriate assessment methods combined with other educational and clinical procedures, could enhance interventions designed to promote the integrated development of individuals from early ages.

## Author Contributions

MN was involved in collecting the data included in this research and had the main responsibility for preparing the manuscript. LR was involved in collecting and analyzing the data. GM was involved in collecting the data. JR was responsible for maintaining the meetings with the schools that participated in the study and conducted advisory work. JL is the main researcher of the study and is responsible for the design of this research, and was involved in preparing the manuscript.

## Conflict of Interest Statement

The authors declare that the research was conducted in the absence of any commercial or financial relationships that could be construed as a potential conflict of interest.
